# Experiences of and recommendations for LGBTQ+-affirming substance use services: A qualitative study with LGBTQ+ people who use opioids and other drugs

**DOI:** 10.21203/rs.3.rs-3303699/v1

**Published:** 2023-09-01

**Authors:** Margaret M. Paschen-Wolff, Avery DeSousa, Emily Allen Paine, Tonda L. Hughes, Aimee N.C. Campbell

**Affiliations:** Columbia University Irving Medical Center and New York State Psychiatric Institute; Columbia University Irving Medical Center and New York State Psychiatric Institute; Columbia University Irving Medical Center and New York State Psychiatric Institute; Columbia University Irving Medical Center and New York State Psychiatric Institute; Columbia University Irving Medical Center and New York State Psychiatric Institute

**Keywords:** bisexual, cultural sensitivity, gay, lesbian, sexual minorities, substance use disorders, substance use treatment, transgender

## Abstract

**Background:**

Lesbian, gay, bisexual, transgender, queer, and other LGBTQ populations (LGBTQ+; e.g., non-binary individuals) have higher rates of substance use (SU) and disorders (SUD) compared to heterosexual and cisgender populations. Such disparities can be attributed to minority stress, including stigma and discrimination in healthcare settings. LGBTQ+-affirming SU treatment and related services remain limited. The purpose of this qualitative study was to characterize LGBTQ + people’s experiences in SU services and recommendations for LGBTQ+-affirming care.

**Methods:**

We conducted demographic surveys (characterized using descriptive statistics) and individual qualitative interviews with N = 23 LGBTQ + people. We employed a flexible coding approach to describe participants’ experiences with stigma, discrimination, and support within SU services; and participant recommendations for how to make such services LGBTQ+-affirming at the patient-, staff-, and organizational-level. We highlighted components of minority stress and mitigators of adverse stress responses throughout our thematic analysis.

**Results:**

Patient-level experiences included bullying, name-calling, sexual harassment, and physical distancing from peers; and support via community-building with LGBTQ + peers. Staff-level experiences included name-calling, denial of services, misgendering, lack of intervention in peer bullying, and assumptions about participants’ sexuality; and support via staff advocacy for LGBTQ + patients, holistic treatment models, and openly LGBTQ + staff. Organizational-level experiences included stigma in binary gendered program structures; and support from programs with gender-affirming groups and housing, and in visual cues (e.g., rainbow flags) of affirming care. Stigma and discrimination led to minority stress processes like identity concealment and stress coping responses like SU relapse; support facilitated SU treatment engagement and retention. Recommendations for LGBTQ+-affirming care included non-discrimination policies, routine pronoun sharing, LGBTQ+-specific programming, hiring LGBTQ + staff, routine staff sensitivity training, and gender-inclusive program structures.

**Conclusions:**

LGBTQ + people experience stigma and discrimination within SU services; supportive and affirming care is vital to reducing treatment barriers and promoting positive health outcomes. The current study offers concrete recommendations for how to deliver LGBTQ+-affirming care, which could reduce SU disparities and drug overdose mortality overall.

## BACKGROUND

Lesbian, gay, bisexual, transgender, queer, and other populations included in the LGBTQ community (LGBTQ+; e.g., non-binary individuals) have higher rates of substance use (SU) and substance use disorders (SUD) compared to heterosexual and cisgender populations.^1–9^ The minority stress framework^10,11^ suggests that LGBTQ + people face unique stressors—e.g., anti-LGBTQ + policies; family rejection; internalized homophobia;^10–12^ and healthcare discrimination ^2^—that can lead to SU as a stress coping mechanism.^13–15^ Without social support and affirming healthcare, SU can progress to SUD.^10–12^ A nationally representative survey of lesbian, gay, bisexual (LGB), and questioning adults found that high vs. low rates of discrimination (including in healthcare settings) doubled the odds of SUD.^14^ In a survey of U.S. transmasculine adults (n = 2,578), 27.6% reported SU in response to discrimination in healthcare settings.^16^

Although only 20% of all U.S. adults who need SU treatment obtain it,^6,17^ stigma and discrimination uniquely reduce SU treatment and healthcare involvement among LGBTQ + people.^2,13,18–21^ Among U.S. transmasculine adults, having been refused medical care based on transgender identity predicted reduced odds of obtaining future care.^16^ LGBTQ + have cited anticipated stigma from providers without LGBTQ+-affirming training as a primary obstacle to utilizing care.^19^ A qualitative study with LGB adults found that LGB-identified or actively LGB-welcoming providers helped alleviate clients’ concerns about sexuality-based stigma.^22^

Despite the value of LGBTQ+-affirming care, the availability of affirming SU treatment is limited. In a study of 446 opioid use disorder (OUD) treatment programs listed in the 2018 Substance Abuse and Mental Health Services Administration (SAMHSA) Treatment Locator as providing “special programs and groups” to LGBTQ + people, only 28% reported offering such services when contacted by phone; fewer still (24%) offered both LGBTQ+-specific services and medications for OUD, the gold standard of OUD treatment.^23^

Numerous studies have identified the need for LGBTQ+-affirming SU treatment.^1,19,24–28^ Since 2014, the U.S. Department of Health and Human Services has designated improved access to LGBTQ+-affirming care as a research priority.^29^ Limited research has centered the voices of LGBTQ + people to guide the development of affirming care. Understanding LGBTQ + experiences with and recommendations for SU-related services is critical to addressing SU disparities and treatment barriers, particularly amid the worsening U.S. drug overdose epidemic.^30^

### Purpose

We conducted individual in-depth interviews with LGBTQ + people who experienced OUD, other SUD, and SU treatment. Our purpose was to: (1) characterize LGBTQ + people’s experiences of (or attitudes about) sexual orientation and gender identity (SOGI)-related discrimination and support in treatment and related services (e.g., 12-step programs; syringe exchange services); and (2) describe interviewees’ recommendations for how to make such services LGBTQ+-affirming. Due to gaps in the literature on opioid use and outcomes among LGBTQ + populations,^31^ the study design focused on LGBTQ + people engaged in illicit opioid use. Ultimately, all participants reported poly-SU and histories of treatment for various SUDs; therefore, this report describes experiences with and recommendations for general SU treatment.

## METHODS

### Participants

The study team was based in New York; however, we recruited participants nationally via: (1) flyers posted within a LGBTQ+-focused health center (just before the COVID-19 pandemic); (2) social media posts (e.g., general posts on Twitter, Facebook, and Instagram; posts within online queer communities); (3) an email via professional listservs to treatment providers in the Tri-state area who shared the study information with their clients; and (4) word of mouth. Eligibility criteria included: (1) age 18 or older; (2) able to speak and understand English; (3) report at least monthly illicit opioid use within the past 12 months or be in recovery for two years or less and used opioids at least monthly within the past two years prior to recovery; and (4) identify as LGBTQ+. Between March and October 2020, we screened 56 potential participants who contacted the study team. Of those screened, 26 were eligible and 23 completed the qualitative interview. We enrolled participants until reaching data saturation. In addition to meeting opioid use eligibility criteria, all enrolled participants reported poly-SU.

### Procedures

Potential participants were instructed to contact the study team via phone, text, or email. By phone, we reviewed a study information sheet, obtained verbal consent., and completed an approximately 10-minute study screener in which we also collected demographic information. Eligible individuals then participated in one-on-one interviews via HIPAA-compliant Zoom video call or phone and received a copy of the study information sheet via email. Interviews lasted approximately 60 minutes (mean = 54 minutes; range: 34 to 100 minutes).

We developed our semi-structured interview guide from extant literature on LGBTQ + barriers to accessing SU treatment services^21,32–35^ and guidelines for affirming SU-related services.^1,19,24–28^ Participants received a digital Visa gift card for $45 via text or email upon interview completion. All interviews were digitally recorded and transcribed by a professional transcription service. The New York State Psychiatric Institute Institutional Review Board approved the study.

### Data Analysis

We used descriptive statistics to characterize the demographic data. Two study authors initially reviewed the first five interviews and discussed key themes to develop the code book. We used a flexible coding approach, in which we initially developed broad “index codes” based on interview guide questions to detect main themes, and subsequently developed more specific analytic codes after determining the focus of study results.^36^ The first and third study author jointly coded three interviews, met to discuss any coding discrepancies, adjusted the index codebook accordingly, and reviewed another three interviews. After reaching intercoder agreement, the lead author index-coded the remaining interviews. The second author assisted with the granular coding for the discrimination, support, and recommendation results; the first and second author met to discuss coding discrepancies across all interviews and to achieve intercoder agreement. We used NVivo 12 Plus to code all interviews.^37^

## RESULTS

### Demographic Characteristics

About half (n = 12) of the 23 participants were from New York; others were spread across the U.S. All other demographic characteristics are reported in Table 1. The mean age was 27.5. All but two participants had at least a high school diploma or GED and most had at least some college. The sample was broadly diverse by race, ethnicity (30% Black, 17% Latinx, 13% multiracial), and SOGI. Over half identified as bisexual or queer, and nearly half identified as transgender or non-binary. Nearly 90% of participants had received formal SU treatment (e.g., inpatient, outpatient, detox). Of the three who had never received treatment, one had participated in a 12-step program and three had received individual therapy. Over 90% of participants reported prescription opioid and/or heroin use in the past 12 months; two participants were in recovery at the time of the interview but reported opioid use within the past two years. Of the 21 participants who had used opioids in the past year, 17 reported daily or near-daily use; the two participants in recovery reported prior weekly use. All 23 participants reported past-12-month poly-SU, including the two in recovery from opioid use, and nearly all reported past-12-month alcohol use (including binge drinking; Table 1).

### Qualitative Interview Themes

We describe participants’ experiences with SU treatment and related services involving SOGI-related discrimination, stigma, and support, as well as participants’ recommendations for how to deliver LGBTQ+-affirming SU services, within three levels of the healthcare system: (1) interactions with peers (patient-level); (2) interactions with staff (staff-level); and (3) organizational policies and structures (organizational-level). Within these levels, we describe components of minority stress (e.g., anticipated and enacted stigma from peers and staff; identity concealment; substance use in response to stigma), as well as supportive environments, groups, and individuals who could mitigate negative impacts of minority stress.^11^ Representative quotes are included within the main text; additional and expanded quotes are in Supplementary Table 1.

### Interactions with Peers (Patient-Level)

#### Discrimination and stigma– overt

Multiple participants reported overt discrimination from peers, including name-calling and homophobic slurs, being misgendered (i.e., referred to with the wrong pronouns), and bullying. For example, one participant (transgender man, age 23, Black) had been called a “he/she and had chips thrown” at him in Alcoholics Anonymous (AA); another (cisgender man, age 33, Black) had been ridiculed at in-patient graduation for kissing and hugging his boyfriend and was subsequently outed on Facebook. The latter participant reported that instances of SOGI-related bullying from peers led to relapse on two separate occasions.

Multiple participants, most of whom were trans-identifying, reported receiving threats of physical violence or sexual harassment from peers in treatment settings. One cisgender gay man’s (age 29, white) roommate left a letter on his bed threatening to lock him in his room at night and force him to perform sexual favors. Another recounted being sexualized by his peers both at an in-patient program and a sober house:

“*It was at that weird place in between my transition where you really can’t tell. That made them uncomfortable because they can’t label me. Or it would be the other end of the spectrum where they would sexualize and fetishize the fact you couldn’t tell... I had a guy make me super uncomfortable because he asked me to have sex with him… Also, at my first sober house… The guys there really sexualized me when they found out that I was into men… They definitely took that as an invite to flirt with me and be openly sexual towards me.*” (transgender man, age 23, white)

#### Discrimination and stigma– indirect

Several participants had also experienced indirect discrimination from other clients, such as visible discomfort or stares from peers.One person noticed peers “scooting away” (cisgender man, age 33, Black) from him in an AA meeting at the sight of his painted nails. One cisgender woman (age 21, Black) described her experience with peers in AA and Narcotics Anonymous (NA): “I’m a fat, black, queer girl, sitting with a bunch of other 18-, 20-year-old white girls that were doing heroin and stuff, and I would try to talk about my experiences, because honestly they were either similar or worse to them, and it was just like no one seemed to even pay attention to what I was saying.”

#### Support– shared life experiences and community-building

Several participants found support and value in “having some sort of shared experience” with LGBTQ + peers in one-on-one interactions and group counseling settings. Only one participant (cisgender woman, age 25, multiracial) had attended an LGBTQ + treatment program, in which educational and social programming like movie nights and dance parties, as well as documentary viewings and workshops on LGBTQ + rights made her feel “comfortable and accepted”. Several other interviewees attended LGBTQ + support groups within general treatment programs, as well as general LGBTQ + and transgender-specific 12-step support groups meetings (e.g., AA). One transgender man (age 21, white) said of his first LGBTQ + AA meeting:

I showed up and there were people wearing drag. There were people wearing leather. Some people were there with their partners and it just seemed like every preconception that I had about AA and God and stuff like had just been shattered at that point.

Participants felt affirmed by LGBTQ + peers who could understand their experiences with SOGI-related discrimination and identity development, and with whom they could center and celebrate LGBTQ + identities and connect over community norms. For instance, one participant (cisgender woman, age 31, Black) noted that “[my LGBTQ + peers and I] would have a text group and people would text you every morning…positive things that had to do with the LGBT community…We all needed that.”

### Interactions with Staff (Staff-Level)

#### Discrimination and stigma– overt

About half the participants experienced overt discrimination from staff while seeking or receiving treatment. For example, two participants stated that providers called them homophobic slurs, one of whom (cisgender man, age 33, Black) also reported two instances of being denied services: once when a nurse at the treatment program refused to assist him, and later while calling local clinics to see if they were LGBTQ+-affirming: “… as soon as I told three of those [programs] that I was openly bisexual, they hung up. I’ve had one tell me to search other…alternative rehab… It felt like what was the point of even trying to get off of drugs if you’re going to have that kind of ignorance from people?”

A few people reported being misgendered or dead-named (i.e., called by a name they no longer use) by staff. A staff member accidentally outed one transgender participant by telling him in the presence of other clients that “it was time to go upstairs…they put me up on the women’s floor because [the staff] claimed it was the only place…that they had a…single room available. And then everybody kind of looked at me like … why do you go up there?... I programmed with the men downstairs. But unless [the other clients] asked, they wouldn’t even know that [I was transgender]” (transgender man, age 21, white, queer). A cisgender woman (age 36, white) reported an instance of a medical provider sexually harassing her by asking invasive questions (e.g., “How did it feel when you were with her?”) exclusively about her female partners.

#### Discrimination and stigma– indirect

Several people reported less overt instances of discrimination from staff, such as non-verbal cues (e.g., therapists whom participants sensed were generally unwelcoming based on their LGBTQ + identities) or tacit behaviors that participants perceived as related to SOGI. Staff often failed to intervene or respond in instances of discrimination or stigma from other clients. For example, one participant’s counselor remained silent when another client called her a homophobic slur in an in-patient AA meeting (cisgender woman, age 31, Black). In other cases, staff actively dismissed or minimized participants’ concerns. One participant stated that after he was physically assaulted by another patient because of his sexual orientation, the program director encouraged him to “… consider choosing a different facility” (cisgender man, age 29, white) instead of taking steps to protect the participant and disciplining the abusive patient. Another participant reported to staff that a patient had sexually propositioned him. When he asked if that patient could be moved to another unit, staff responded, “you’re both being discharged in a couple days, it will be fine” (transgender man, age 23, white, queer).

In other instances, participants carried the burden of addressing discrimination and stigma from other clients, rather than staff members taking on that responsibility. This was evident in one participant’s description of a session with their intensive outpatient counselor:

“*I was expressing my frustration of being misgendered. People would call me [by the correct name] but then they would use ‘she/her’ pronoun stuff. And I just didn’t feel [that my counselor] was very supportive of that. She was just kind of like, ‘You should bring it up’ … And I guess that part of it might have been that she was trying to empower me or something, but it just felt a little lonely.*” (transgender man, age 28, Latinx)

#### Discrimination and stigma– assumptions about SOGI

In addition to lacking staff support, a few respondents also encountered assumptions from staff about their sexual identity or behavior, such as staff presuming clients’ sexual identity labels based on their reported sexual history rather than asking clients to self-label, or staff profiling LGBTQ + clients as more “promiscuous” than their non-heterosexual peers. For example, when one participant (transgender man, age 38, Latinx) told his provider that he was married to a woman and attracted to men, the provider “… [implied] that [I was] a cheater or that I’m lying to myself or someone else, when clearly, I’m being completely out with everything.”

Two participants’ providers suggested that participants would want to sleep with their peers based on their sexual identity. In one case, a provider assumed that a participant and his close friend were in a relationship “… because two people are LGBT, they obviously have to be interested in each other. So, they were trying to separate us and put us as far away from each other as they could” (cisgender man, age 29, white). Another respondent (transgender man, age 23, white) noted: “… it was heavily insinuated by staff that I couldn’t be left alone with any - I had to have eyes on me, like one on one because in my health chart it says, ‘high risk sexual behavior.’” One participant (transgender man, age 28, Latinx) reported having to educate their therapist who “… sometimes [will] assume about my experience based on another client of his who’s trans .... there is some commonality to our experiences, but we’re all individual people too.” Finally, although several participants made a connection between SOGI-related trauma, LGBTQ + identity, and SU, one participant cited his transgender identity as a positive aspect of his life and feeling “frustrated” with past therapists who assumed that it caused his addictions.

#### Discrimination and stigma– absence of direct SOGI discussions

By contrast, several participants stressed that treatment address the connection between LGBTQ + experiences and addiction, with one person (transgender man, age 28, Latinx) noting that his gender identity “influences…why [he] started using.” The lack of SOGI discussions in treatment often created a less welcoming environment. For example, one participant (cisgender man, age 29, white) said that while discussions about SOGI were not outwardly discouraged within his inpatient program, “[he] definitely didn’t feel comfortable talking about it.” Another participant (cisgender woman, age 21, Black) reported that when she brought up her sexual orientation in treatment, providers moved on to other topics.

#### Discrimination and stigma – Identity concealment response

Due to either experiencing or anticipating SOGI-related discrimination and stigma, a few participants reported concealing their identities in SU treatment settings. After a negative first experience seeking treatment led to a relapse, one participant (cisgender man, age 33, Black) attempted to conceal his orientation during his next treatment experience: “Maybe if I hide [my bisexuality] for as long as I can, I can get some kind of treatment for a while and maybe… I won’t relapse.” Another respondent (cisgender woman, age 25, multiracial) reported that although staff in her inpatient program did not say anything outwardly discriminatory, the fact that the environment was not explicitly welcoming to LGBTQ + people (e.g., no LGBTQ + representation in their reading materials) meant that she “couldn’t really be [herself]” within the program.

#### Support – advocacy for LGBTQ + clients

For many participants, SOGI-related support also came from staff members. Some described staff members who had advocated on their behalf, either with other staff or on a more personal level. For example, one person (cisgender man, age 33, Black) discussed a nurse who briefed the rest of the clinical team on his genderfluidity and bisexuality, which helped assuage staff nervousness about how to approach the participant and lead to more respectful interactions with staff : “And after [the nurse] said that, the nurses who had just come in, ‘How are you feeling today? Are you okay, sir, ma’am?’ Or, they would ask me, you know, ‘What do I want to be called?’”

For more than half of participants, the SOGI-related support they received from SU treatment staff was often “hand in hand with…instances of discrimination”. For instance, one participant (cisgender man, age 29, white) noted that “the trauma counselor…was helping me stay away from a client who was discriminating and kept me closer to [another client] that I connected with and was able to talk to.” Another participant (transgender man, age 33, Black) recalled a counselor who allowed him to use her personal restroom after other patients were upset that he was using the men’s room.

##### Support – LGBTQ+-identifying and allied providers

A few participants identified providers who were openly LGBTQ + or well-versed in LGBTQ + issues as important sources of support who made participants comfortable being open about their own identity in treatment: “…[The counselor] opened up with jokes about being a lesbian and it just made me right then feel that I don’t have to hide who I am. There are people like me. I’m not weird. I’m not funny. I’m not whatever people think I am, and I think that just was very supportive” (cisgender woman, age 25, multiracial). Others appreciated that their providers understood their SOGI, and the connection between LGBTQ+-related trauma and addiction, without the client having to explain this. For example, one participant (non-binary, age 31, multiracial) described a counselor who “understood exactly what I meant” when the participant described having a girlfriend and also flirting with a man.

### Experiences with Organizational Policies and Structures (Organizational-Level)

#### Discrimination and stigma – gendered program structures

Participants also described experiences with discrimination and stigma at the institutional level. A few participants commented on the binary, gendered nature of treatment programs across the board, from AA meetings to sober houses to in-patient programs: “There are definitely gendered [AA] meetings, as well as all sober houses are gender segregated. There’s no trans or queer sober houses. They are all either men or women. I’ve always been in men’s houses, and I’ve always been the only trans person” (transgender man, age 23, white). One person, who identified as nonbinary (age 29, Black, queer), spoke about programs that will “only accept men patients or women patients.” Two others reported being assigned to a room according to their sex assigned at birth, rather than their gender identity.

#### Support – gender-affirming program structures

By contrast, a few participants recalled being assigned to rooms that aligned with their gender identity or were otherwise provided with the option. One participant (transgender man, age 21, white) in an outpatient program that had opened gender-inclusive housing noted that the program asked “where [he] was most comfortable being housed” and appreciated that “my gender and sexuality…wasn’t really a focus…I like just being able to be integrated in with everybody else.” This participant was also the only person who explicitly mentioned a SOGI-related non-discrimination policy within a treatment program. A few other participants also described intake processes that were particularly inclusive of LGBTQ + people, such as staff explaining the need for questions about sex assigned at birth and including expansive and open-ended response options for sexual identity on intake forms.

#### Support – affirming treatment environment

While several participants experienced supportive policies and procedures, only two noted aspects of the physical environment that were specifically supportive of LGBTQ + clients. These included LGBTQ+-specific signage, rainbow flags, supportive messages, and drawings created by the clients themselves.

Several participants also appreciated programs that took a non-judgmental, harm reduction approach, which they often encountered in syringe exchange programs. Syringe exchange programs prioritized safety above all else, had a “no questions asked” attitude, and were particularly welcoming environments for transgender people. For example, one participant (transgender man, age 28, Latinx) described a syringe exchange program as follows:

They truly don’t care who you are, your background, anything like that. They just want to keep you safe and that’s it…the one here especially is really good about trans issues because they also do a lot of outreach and there are a lot of trans women involved in that. So, they’re really, really good about being respectful of people’s genders…

Although most participants described specific experiences with SOGI-related discrimination and stigma with SU treatment settings, six participants noted that they had never encountered SOGI-related mistreatment.

#### Recommendations for LGBTQ+-Affirming SU Treatment and Services

Based on their lived experiences with addiction and SU treatment and services, participants made recommendations for how to make services LGBTQ+-affirming. Table 2 includes an outline of these recommendations.

#### Patient-level recommendations

Participants recommended that SU treatment programs have clearly documented non-discrimination policies to address SOGI-related discrimination and stigma from peers when it did occur. Policies should include formal guidelines on disciplinary actions for peers who mistreat LGBTQ + clients, such as tiered responses based on the severity of the mistreatment (e.g., name-calling versus threats of sexual assault). Participants also suggested that policies routinize the process of peers sharing their names and pronouns as part of introductions within treatment settings. This could help mitigate misgendering and dead-naming from peers, set a tone of inclusivity for all clients, and reduce stigma toward transgender and non-binary clients.

Drawing on their positive experiences with LGBTQ + peers, several participants suggested that LGBTQ+-exclusive treatment programs “could be very helpful in bringing a community together and getting people some help that maybe they wouldn’t get in another program” (cisgender man, age 29, white). Others recommended that programs for the general population continue to offer LGBTQ+-specific groups. Several other participants expressed ambivalence: on the one hand, having broadly inclusive programs with LGBTQ + clients integrated into the general patient population could decrease othering of and stigma toward LGBTQ + people; on the other hand, LGBTQ+-exclusive programs and groups could create a sense of safety.

#### Staff-level recommendations

Like patient-level recommendations, several participants also suggested that SU treatment programs develop formal non-discrimination policies and disciplinary guidelines to address staff mistreatment of LGBTQ + clients. Participants also recommended that programs institute policies around vetting staff for LGBTQ+-affirming views and practices and should promote the hiring of openly LGBTQ + staff at all levels, from support staff to behavioral health providers to medical doctors. One participant (non-binary, age 29, Black, queer) said that “…if the place is going to say that they’re going to cater to LGBTQ people, then the people that are working there [should] actually reflect that.”

Participants made recommendations for providers to deliver LGBTQ+-affirming treatment sessions, including asking clients’ pronouns and sharing their own pronouns in session, asking about and exploring SOGI beyond the intake process, and continuing to understand clients holistically rather than focusing only on their gender and sexuality. Several participants noted that treatment should foster patients’ exploration of the connection between addiction, SOGI-related trauma, discrimination, and stigma.

Nearly all participants recommended that staff receive sensitivity training around working with LGBTQ + clients, speculating that staff may be “scared”, “nervous”, “afraid”, or even “phobic” around discussing SOGI and inadvertently making offensive statements. Training would thus need to account for and address staff members’ fear and anxiety. Recommended training content included how to avoid making SOGI-related assumptions based on the gender of clients’ partners, how to respectfully ask clients about SOGI, education on SOGI-related language and terminology (e.g., what does the LGBTQ + acronym stand for; slang terms), and information about LGBTQ + communities (e.g., chosen families; the importance of LGBTQ+-affirming social spaces). Nearly half the participants suggested that staff receive specific training on working with transgender and gender diverse clients (e.g., normalize sharing and asking about pronouns and correct names). Additionally, participants suggested that staff receive guidance on how to correct instances of misgendering without further “alienating” clients:

*“I would say [staff trainings are] one of the most important things. Having people who are queer competent…so they know how to refer to someone and how to correct themselves. That’s one of the biggest things, when I correct someone on my pronouns, I don’t want them to be like, ‘OMG I’m so sorry’. Then I feel like I have to calm them down and say it’s okay, when that’s really not my job... They should be trained to be like okay, I will adjust my language and leave it at that… It won’t happen again… Just to make sure the queer people don’t feel more alienated than they already do.”* (transgender man, age 23, white)

A few participants also indicated a need for staff training on how to intervene when LGBTQ + clients experience discrimination, stigma, or aggression from other clients. For example, staff should receive guidance on whether to step in when clients use the wrong pronouns for other clients, and on how to address sexual harassment of LGBTQ + clients so that LGBTQ + people don’t feel like they are “kind of pushed aside because the staff is afraid of handling it” (transgender man, age 21, white). Several participants highlighted that staff should be trained to consider clients’ intersecting marginalized identities (such as LGBTQ + people of color) and cultural backgrounds and how experiences related to those identities may be interconnected with addiction. Several participants similarly noted that staff should receive training on how LGBTQ + people have unique experiences with discrimination and stigma (i.e., minority stress), including direct harassment from other people as well as discriminatory policies and laws. One participant (cisgender woman, age 31, white) suggested that not only should staff receive training in these areas, but SU treatment programs should also actively show support for LGBTQ + communities by participating in rallies and LGBTQ + pride marches.

Finally, several participants gave recommendations on the format and frequency of staff training, from once a year to once a month. Other participants suggested that training should be interactive and potentially spaced out over a few days. Two participants noted that training would need to be “individualized”, and the format, frequency and comprehensiveness should depend on the staff member’s role and level of contact with patients (e.g., trainings might be different for a driver vs. a behavioral health provider). A few participants stated that people who identify as LGBTQ+, such as those who had struggled with SUD, should deliver staff trainings.

#### Organizational-level recommendations

To reinforce LGBTQ+-affirming care at the organizational level, participants made recommendations about program policies, intake forms and processes, and visual cues of affirmation to include in the physical environment. Two participants recommended that LGBTQ + people “have a voice” in the development of non-discrimination and staff vetting policies within SU treatment programs by meeting with program staff and directors. Some participants noted that non-discrimination policies should specifically address gender-affirming care, including guidelines on the use of clients’ correct names and pronouns and relaxing dress code policies. Other participants thought programs might be required to ask about sex assigned at birth and names on documents for legal and health insurance reasons but suggested that programs develop policies and procedures for how to sensitively explain this requirement to clients.

Although many participants shared recommendations on programmatic policies, several others weren’t sure what to suggest. A couple participants noted that many SU treatment programs do not admit openly LGBTQ + patients in the first place, so focusing on non-discrimination policies may not be the best starting point for promoting LGBTQ + affirming care. One participant (cisgender woman, age 21, Black) stated that “…it doesn’t matter what the policy is in your program if [LGBTQ + clients are] not in it.”

Many participants recommended that intake forms include questions about the name they go by, pronouns, and gender identity, especially because clients’ current name might differ from the name on their legal documents. Many participants suggested that rather than having check boxes with pre-written SOGI responses, forms should instead “have lines to write on…and I understand it’s harder for data collection. But someone else can figure out how to make that work… I think that the more you can let people self-identify, self-disclose, actually the more, better information you get” (transgender man, age 38, Latinx).

For programs that do include check boxes, several participants recommended having response options beyond “male” and “female”, or “straight”, “lesbian”, “gay”, and “bisexual” since some participants may not use any of these more binary, rigid labels. Having more expansive options could not only make LGBTQ + people feel more welcome but could also help ease the stress of initiating SU treatment. On the other hand, some participants suggested that sharing one’s SOGI at intake should be voluntary due to the sensitive nature of such questions. These participants talked about the need for balance between asking intake questions that demonstrate a program’s comfort with discussing SOGI and avoiding questions that could feel irrelevant to the treatment or overly personal.

Nearly half the participants stated that visual cues in the physical environment would signal that programs are welcoming to LGBTQ + people. For example, many participants noted that having a rainbow flag somewhere within the organization would assuage staff anxiety about raising SOGI-related topics in the treatment setting. Several participants recommended that programs hang posters of LGBTQ + couples and transgender and non-binary people related to SU treatment with “motivational” messages. Two participants proposed that programs show videos with scenarios about substance use that include racially diverse LGBTQ + characters. Some participants also suggested that more discreet signifiers like videos, brochures, and small rainbow flags would go a long way, even if the program was not LGBTQ+-specific.

One participant (cisgender man, age 29, white), however, noted that simply hanging a rainbow flag doesn’t indicate whether an organization integrates LGBTQ+-affirming practices throughout their program: “…this place I went to did have a rainbow flag hanging up to I guess state that [they were LGBTQ+-affirming], but didn’t necessarily make me feel more comfortable there. Like you could say, you support this, but it doesn’t necessarily mean that there’s any follow through.” Some participants suggested concrete steps that programs could take to be truly LGBTQ+-affirming, such as educating the public on LGBTQ + issues, hosting LGBTQ + events, and connecting transgender people with affirming services such as hormone replacement therapy and name change clinics.

In addition to recommendations for visual cues of LGBTQ+-affirming spaces, several participants also had suggestions for how spaces should be structured to affirm clients’ gender identity. Some participants acknowledged the nuances in creating safe spaces for transgender people without making them feel excluded. Some felt that people should be encouraged to use “whatever [gender-segregated] bathroom you’re comfortable using” and should be housed based on gender identity or in gender-neutral spaces, rather than having separate bathrooms and living spaces for LGBTQ + people. As one transgender man (age 28, Latinx, gay) summed it up:

…it’s hard because you don’t want to be singled out as trans person but also you want to make sure that you feel safe. Because I don’t know that I would want to room with a cis[gender] man either. So maybe like having individual rooms that LGBT people can maybe opt into; because I could also see a cis[gender] gay man not wanting to room necessarily with a straight guy depending on who that is…It’s very easy to make gender neutral bathrooms.

## DISCUSSION

This qualitative study describes the lived experiences of 23 racially and geographically diverse LGBTQ + people in OUD treatment and other related services. Most participants had experienced some form of SOGI-related discrimination or stigma in these settings, and most had also received SOGI-related support. Participants experienced discrimination and support from peers, staff, and at the organizational level. Based on these lived experiences, participants made recommendations for SU treatment programs and services to reduce discrimination and stigma and increase support for LGBTQ + clients.

### SU Treatment/Services Experiences and Recommendations: Patient-Level

Discriminatory experiences with peers included bullying, name-calling, sexual harassment, threats of physical violence, visible discomfort, and physical distancing. Stigma from peers led to relapse for one participant, demonstrating a stress coping response as described within the minority stress framework.^11^ Peer-related support included discussing shared experiences with SU, LGBTQ+-related discrimination, and LGBTQ + identities both one-on-one and in group settings. Participants recommended that programs develop policies for addressing and disciplining discriminatory peers, make the sharing of pronouns a routine part of peer-to-peer introductions, and offer LGBTQ+-specific programming within treatment to create a sense of community and increase opportunities for sharing common experiences.

Research has found that peer support within SU treatment promotes positive treatment outcomes, such as lower rates of SU relapse, more positive interactions with treatment providers, ongoing treatment engagement, and increased satisfaction with treatment.^38,39^ The minority stress framework also suggests that being part of an LGBTQ + community can help mitigate the negative impact of discriminatory experiences.^11^ LGBTQ+-specific groups and community events within treatment are also crucial to improving treatment outcomes and are included in SAMHSA’s guiding principles for SU treatment with LGBTQ + clients;^40^ however, barriers to providing and accessing such services include limited funding, not enough LGBTQ + clients to form groups,^25^ and incorrect advertising of specialized LGBTQ + SU programming.^23^ Future federal funding should be allocated to support LGBTQ+-specific SU treatment, and SAMHSA should consider re-designing its treatment program survey to more accurately identify SU treatment programs tailored to LGBTQ + communities.^23^ Further, given that research on the impact of peer discrimination in SU treatment remains limited, future research should further explore how such experiences impact treatment outcomes among LGBTQ + clients and consider how to mitigate the negative effects of peer-based discrimination.

### SU Treatment/Services Experiences and Recommendations: Staff-Level

Like their experiences with peers, participants also reported overt, direct discrimination from staff such as SOGI-related name-calling, dead-naming, and misgendering, as well as denial of services. Participants further contended with general coldness from providers, lack of intervention in instances of bullying from peers, providers’ stigmatizing assumptions about participants’ sexual identity and behavior, and lack of direct discussions about SOGI within treatment. Stigma from providers lead to denial of services and SOGI concealment in some cases, which are mechanisms of minority stress processes.^11^

Participants also encountered staff who advocated on their behalf and were sensitive to how SOGI-related trauma was interrelated with SU; and openly LGBTQ + staff who were particularly supportive and relatable. As a result of these experiences, participants recommended that programs develop non-discrimination policies to hold staff accountable and hire LGBTQ + providers and allies; that staff treat patients holistically and explore connections between SOGI-related discrimination and SU; and perhaps most importantly, that staff receive routine training on how to provide LGBTQ + affirming care.

Previous research has highlighted the importance of reducing discrimination and stigma from providers in healthcare settings to reduce the problematic SU and SUD as stress coping responses.^14,16,41^ Minimizing provider-based discrimination^16,19^ and hiring LGBTQ+-affirming providers—whether LGBTQ+-identifying or strong allies^19,22^—can also promote treatment initiation and improve retention among LGBTQ + people. Research has also documented the need for training staff in providing gender-affirming care and addressing LGBTQ + mental health needs to reduce barriers to treatment;^19,27^ SAMHA’s 2012 treatment guidelines reinforce the importance of staff training for promoting LGBTQ+-affirming care, as well as participants’ recommendations that providers treat patients holistically and account for intersectional identities.^40^ Trainings for providers should therefore use an intersectional lens that highlights the diversity of LGBTQ + communities, as well as how intersecting marginalized identities may compound SOGI minority stress responses like SU.^42,43^ Previous research also echoes participants’ sentiments in the current study that providers should avoid pathologizing LGBTQ + patients and focusing only on SOGI without considering other identities.^21^ As the participants in the current study have noted, trainings should be comprehensive and occur at least yearly to ensure that staff are updated on and reminded of principles of LGBTQ+-affirming care.

### SU Treatment/Services Experiences and Recommendations: Organizational-Level

At the organizational level, participants described feeling stigmatized within treatment and 12-step programs structured around binary gender identities, but support from other programs that offered gender-affirming and inclusive groups and housing. Some participants felt supported by explicitly welcoming visual cues like rainbow flags and LGBTQ+-specific imagery. Again, such supportive environments can alleviate the negative effects of minority stress experiences and reduce adverse outcomes^11^ like SU, relapse and overdose. Participants cited programs like syringe exchanges as particularly welcoming and supportive in their non-judgmental, harm reductionist, all-inclusive orientation. Participants described a harm reduction approach that prioritized safety as especially LGBTQ+-affirming given that clients were not required to reveal overly personal aspects of their lives like SOGI or legal names to receive services. These findings suggest that while asking about SOGI within SU service settings can reduce stigma and make LGBTQ + people feel seen, services that have a “no-questions-asked” policy can also be affirming.

To enhance LGBTQ+-affirming SU treatment and services at the organizational level, participants recommended that LGBTQ + clients have a voice in treatment policy development, and that policies include guidelines on the provision of gender affirming care. Participants also suggested that intake forms ask about correct names, pronouns, and expansive SOGI identities with write-in and voluntary response options. Participants advised that programs continue to include visual indicators of being LGBTQ+-affirming, including flags, motivational messages, and inclusive waiting room videos; programs would also need to actively implement explicitly affirming policies and procedures beyond such visual cues. Finally, participants recommended that programs either provide single-stall gender-neutral bathrooms or allow clients to use the bathroom aligned with their gender identity.

Prior research has stressed that a gender-affirming treatment environment is crucial to improving treatment engagement among transgender SU treatment clients.^44^ Participants’ recommendations are also in line with previous literature stressing the importance of including SOGI questions—particularly those that are open-ended—on intake forms to foster LGBTQ+-affirming interactions throughout treatment and avoid pitfalls like assuming clients’ family structures.^21^ SAMHSA’s SU treatment guidelines also echo participants’ recommendations that programs create a welcoming environment with physical indicators of LGBTQ + inclusivity.^40^ To ensure that LGBTQ + people are actively included in developing and guiding SU treatment policies and procedures, SU treatment programs and services could develop patient or community advisory boards comprised of LGBTQ + with lived SU experience. Additional research could also further explore how to link organizational structures that set the stage for LGBTQ+-affirming SU treatment and the delivery of such treatment from staff members.

### Limitations

The current study is not without limitations. First, findings may be weakened by self-report bias, and by the fact that some participants were recounting experiences from several years prior to the interview. Second, although nearly half of participants were in various states, just over fifty percent were in New York State, which may indicate a biased perspective on SU treatment experiences. Third, despite our recruitment efforts, we enrolled a relatively small sample, which could introduce self-selection bias as to individuals who may have chosen to screen and enroll in the study. Moreover, none of the participants identified as transgender women; thus, our findings do not represent the experiences of transgender women. The sample was also largely young (the oldest participant was 38 years old; mean age was 28), thus limiting our ability to report on SU treatment experiences of older LGBTQ + adults. Additionally, data were collected in the early months of the COVID-19 pandemic, when there were extensive disruptions in SU treatment and other related services, as well as rising rates of SU among LGBTQ + populations.^45,46^ Thus, the timing of the data collection could influence the study findings. These limitations point to important areas for future research— for example, the experiences of transgender women and older LGBTQ + adults within SU treatment.

## CONCLUSIONS

The current study demonstrates that LGBTQ + people continue to experience discrimination and stigma within SU services at multiple levels, including from peers, providers, and organizational structures; yet many LGBTQ + individuals also experience support from the same sources. Such discrimination can exacerbate minority stress processes like identity concealment, and stress coping responses like SU relapse, while support can assuage negative outcomes of minority stress and facilitate treatment engagement and retention. Based on their experiences with SU programming, the participants in the current study recommended a range of strategies to promote affirming care, including non-discrimination policies, LGBTQ+-specific and gender-affirming programming, rigorous staff training, and direct LGBTQ + client involvement in SU treatment planning and policy making. This study offers new information since the decade-old SAMHSA guidelines on LGBTQ+-affirming SU treatment, a decade in which LGBTQ + people (along with the general population) have experienced a global pandemic, rising overdose mortality, and increasingly anti-LGBTQ + laws and policies. SU treatment programs should consider adopting these recommendations to ensure that LGBTQ + people receive support and affirmation, which could close gaps in SU treatment access, reduce risk of drug overdoses, and promote overall health of LGBTQ + people in a time of significant social and political upheaval.

## Figures and Tables

**Table 1:** Demographic characteristics of N = 23 LGBTQ + participants

	Range	Mean (SD)
Age in years	21–38	27.5 (5.1)
	*n*	%
Education		
Primary school/some high school	2	8.7
High school diploma/GED	7	30.4
Some college, no degree	3	13.0
Associate degree	2	8.7
Bachelor’s degree	8	34.8
PhD, MD, or other doctoral degree	1	4.4
Race & Ethnicity		
Black/African-American, non-Hispanic	7	30.4
Latinx, any race	4	17.4
Multiracial, non-Hispanic	3	13.0
White, non-Hispanic	9	39.1
Sexual identity		
Bisexual	5	21.7
Gay	4	17.4
Lesbian	4	17.4
No preference	1	4.4
Pansexual	2	8.7
Queer	7	30.4
Gender identity		
Cisgender man	4	17.4
Cisgender woman	8	34.8
Non-binary	5	21.7
Transgender man	6	26.1
Formal substance use (e.g., inpatient, outpatient, detox) treatment experience	20	87.0
Past 12-month substance use		
Poly-substance use	23	100.0
Alcohol	22	95.65
Opioids ^a^ (e.g., prescription ^b^, e.g., Oxycontin, Vicodin, Percocet], heroin, fentanyl)	21	91.30
Cannabis	14	60.87
Ecstasy	12	52.17
Hallucinogens (e.g., LSD, mushrooms)	11	47.83
Prescription^a^ sedatives (e.g., Valium, Ativan, Xanax, Klonopin)	9	39.13
Prescription^a^ stimulants (e.g., Adderall, Ritalin)	8	34.78
Poppers	6	26.09
Injection drug use (any drug)	6	26.09
Powered cocaine	5	21.74
Crack cocaine	4	17.39
Crystal methamphetamine	4	17.39
Ketamine	4	17.39
Other drugs (i.e., Gabapentin; K2 spice)	3	13.04
GHB	1	4.35
Past 12-month substance use frequency		
Binge alcohol use ^c^	22	
*Daily/almost daily*	10	45.45
*Weekly*	7	31.82
*Monthly*	0	0.00
*Less than monthly*	5	22.73
Opioid use	21	
*Daily/almost daily*	17	80.95
*Weekly*	3	13.64
*Monthly*	1	4.55
*Less than monthly*	0	0.00

a Two participants were in recovery at the time of the interview but had used opioids prior to recovery (within the last two years)

b Used other than as prescribed by a doctor, e.g., higher quantity or frequency than prescribed, someone else’s prescription

c Assigned female at birth = 4 or more drinks in one sitting; assigned male at birth = 5 or more drinks in one sitting

**Table 2:** Recommendations from N = 23 LGBTQ + people with lived experience on the provision of LGBTQ+-affirming SU treatment and services

Patient-Level Recommendations	

Patient-Related Policies	• Develop and clearly document SOGI-related non-discrimination policies, including guidelines on disciplinary actions for peers who mistreat LGBTQ + clients with tiered responses based on severity of mistreatment (e.g., name-calling vs. sexual assault).
	• Develop policies on the routinization of peers sharing names and pronouns as part of introductions.

LGBTQ+-Specific Services to Create Community and Safe Space	• Where possible, create LGBTQ+-exclusive SU treatment programs. If not possible, offer LGBTQ+-specific groups within larger SU treatment programs and ensure that programs are broadly inclusive of LGBTQ + clients beyond specific groups.

**Staff-Level Recommendations**	

Staff-Related Policies	• Develop and clearly document SOGI-related non-discrimination policies, including guidelines on disciplinary actions for staff members who mistreat LGBTQ + clients.
	• Develop policies to vet staff for LGBTQ+-affirming views and practices.

Staff Hiring	• Hire openly LGBTQ+ (or explicitly LGBTQ+-allied) staff at all levels, from support staff to behavioral health providers to medical doctors.

Provision of Affirming Treatment	• Staff should share their pronouns and ask clients to do so within group and individual treatment sessions.
	• Staff should guide patients in exploring the connection between addiction, SOGI- related trauma, discrimination, and stigma, while also recognizing that identifying as LGBTQ + does not always lead to trauma or negative health outcomes.
	• Staff should view LGBTQ + clients holistically (including accounting for unique experiences based on racial, ethnic, and other salient identities) rather than only focusing on SOGI.

Staff Training	• Staff should receive LGBTQ + sensitivity training that accounts for fear and anxiety about to work with LGBTQ + clients. Training content should guide staff in how to avoid making SOGI-related assumptions about clients based on the gender of clients’ partners, how to respectfully ask clients about SOGI, education on SOGI-related language and terminology (what does the LGBTQ + acronym stand for; slang terms), information about LGBTQ + communities (e.g., chosen family; importance of LGBTQ+-affirming social spaces), and guidelines for working with transgender and gender diverse clients (e.g., normalize sharing and asking about pronouns and correct names).
	• Staff should receive training in how to intervene when LGBTQ + clients experience discrimination, stigma, or aggression from other clients.
	• Staff training should use an intersectional lens to teach staff how to effectively work with clients with intersecting marginalized identities (such as LGBTQ + people of color).
	• Trainings should occur regularly (e.g., at least once a year) and should be interactive and individualized based on staff role (e.g., medical doctors, mental health providers, receptionists).
	• People who identify as LGBTQ + should deliver staff trainings.

**Organizational-Level Recommendations**	

Organizational Policies	• Include LGBTQ + people in the development of non-discrimination and staff vetting policies by forming community advisory boards and meeting with LGBTQ + people with lived experiences in addiction and SU treatment and other services.
	• Non-discrimination policies should address gender affirming care, including guidelines on the use of clients’ correct names and pronouns and relaxing dress code policies.

Intake Forms and Processes	• Intake forms should include questions about the name participants go by, pronouns, gender identity, and sexual orientation, with options for open-ended responses as well as pre-written response options beyond “male” and “female”; “straight”, “lesbian”, “gay”, and “bisexual”.
	• Responses to SOGI intake questions should be voluntary, recognizing the potentially sensitive nature of such questions.

Visual Cues of an Affirming Environment	• Display rainbow flags; LGBTQ+-specific brochures, signs, and banners (e.g., posters with LGBTQ + couples and transgender and non-binary people related to SU treatment with motivational messages); and videos with scenarios about SU that include racially diverse LGBTQ + characters.
	• Ensure that programs are affirming beyond visual cues (e.g., educating the public on LGBTQ + issues, hosting LGBTQ + events, and connecting transgender people with affirming services such as hormone replacement therapy and name change clinics).

Structure of Programs Related to Gender Identity	• Ensure that programs are safe spaces for transgender and non-binary people, e.g., by offering single-stall gender-neutral bathrooms or by allowing people to use the bathroom aligned with their gender identity; and by offering gender-neutral living arrangements or housing people based on their gender identity.

NOTES: LGBTQ + = Lesbian, gay, bisexual, transgender, queer, and other populations within the LGBTQ community (e.g., non-binary individuals); SOGI = sexual orientation and gender identity; SU = substance use

## List Of Abbreviations

AA: Alcoholics Anonymous
LGB: Lesbian, gay, and bisexual
LGBTQ+: Lesbian, gay, bisexual, transgender, queer, and other populations within the LGBTQ community (e.g., non-binary individuals
NA: Narcotics Anonymous
OUD: Opioid use disorder
SOGI: Sexual orientation and gender identity
SAMHSA: Substance Abuse and Mental Health Services Administration
SU: Substance use
SUD: Substance use disorders
